# Application of Bayesian structural equation modeling in construction and demolition waste management studies: Development of an extended theory of planned behavior

**DOI:** 10.1371/journal.pone.0290376

**Published:** 2024-01-23

**Authors:** Nur Anisah Mohamed, Ayed R. A. Alanzi, Azlinna Noor Azizan, Suzana Ariff Azizan, Nadia Samsudin, Hashem Salarzadeh Jenatabadi

**Affiliations:** 1 Institute of Mathematical Sciences, Faculty of Science, Universiti Malaya, Kuala Lumpur, Malaysia; 2 Department of Mathematics, College of Science and Arts in Gurayat, Jouf University, Gurayat, Saudi Arabiai; 3 College of Business Administration, Prince Sultan University, Riyadh, Saudi Arabiai; 4 Department of Science and Technology Studies, Faculty of Science, Universiti Malaya, Kuala Lumpur, Malaysia; 5 Faculty of Social Sciences and Liberal Arts, UCSI University, Kuala Lumpur, Malaysia; Universiti Utara Malaysia, MALAYSIA

## Abstract

Sustainable construction and demolition waste management relies heavily on the attitudes and actions of its constituents; nevertheless, deep analysis for introducing the best estimator is rarely attained. The main objective of this study is to perform a comparison analysis among different approaches of Structural Equation Modeling (SEM) in Construction and Demolition Waste Management (C&DWM) modeling based on an Extended Theory of Planned Behaviour (Extended TPB). The introduced research model includes twelve latent variables, six independent variables, one mediator, three control variables, and one dependent variable. Maximum likelihood (ML), partial least square (PLS), and Bayesian estimators were considered in this study. The output of SEM with the Bayesian estimator was 85.8%, and among effectiveness of six main variables on C&DWM Behavioral (Depenmalaydent variables), five of them have significant relations. Meanwhile, the variation based on SEM with ML estimator was equal to 78.2%, and four correlations with dependent variable have significant relationship. At the conclusion, the R-square of SEM with the PLS estimator was equivalent to 73.4% and three correlations with the dependent variable had significant relationships. At the same time, the values of the three statistical indices include root mean square error (RMSE), mean absolute percentage error (MPE), and mean absolute error (MSE) with involving Bayesian estimator are lower than both ML and PLS estimators. Therefore, compared to both PLS and ML, the predicted values of the Bayesian estimator are closer to the observed values. The lower values of MPE, RMSE, and MSE and the higher values of R-square will generate better goodness of fit for SEM with a Bayesian estimator. Moreover, the SEM with a Bayesian estimator revealed better data fit than both the PLS and ML estimators. The pattern shows that the relationship between research variables can change with different estimators. Hence, researchers using the SEM technique must carefully consider the primary estimator for their data analysis. The precaution is necessary because higher error means different regression coefficients in the research model.

## Introduction

Every year, the amount of waste that is produced as a result of construction and demolition (C&D) activities in the world is greater than 10 billion tonnes [1]. It has been a significant obstacle for nations all over the world to effectively manage the waste that they generate and to lessen the negative effects of the waste. On the one hand, the generation of construction and demolition waste is responsible for a wide variety of unfavourable effects, including the wastage of raw materials, the use of energy, and the polluting of water and land. In addition to this, the treatment of the waste would demand a large amount of both financial and labour resources [2, 3]. On the other side, waste management and reduction can bring a number of benefits, including a reduction in the amount of garbage dumped in landfills and a lower consumption of raw materials [4, 5].

In these days, construction and demolition activities create the most significant worldwide waste stream [6]. Growing population and urbanization also significantly caused the generation of C&DW worldwide [7]. Some countries are trying to reduce C&DW. In China, Shanghai and Shenzhen are the best cities that have accomplished more than 15% recovery rates [8]. However, a current study by Ma, Tam [9] mentioned that this number is still meaningfully less than few developed countries, such as 70% of the United States, 88% in Germany, and 96% in Japan. C&DW constitutes a majority of the total waste dumped in landfills [10]. C&DW in landfills containing concrete, metals wood, bricks, glass, plastic, gypsum, solvents, asbestos and excavated soil [11]. Ajayi, Oyedele [12] mentioned that the global average of landfilled C&D waste is about 35%.

**Fig 1:**
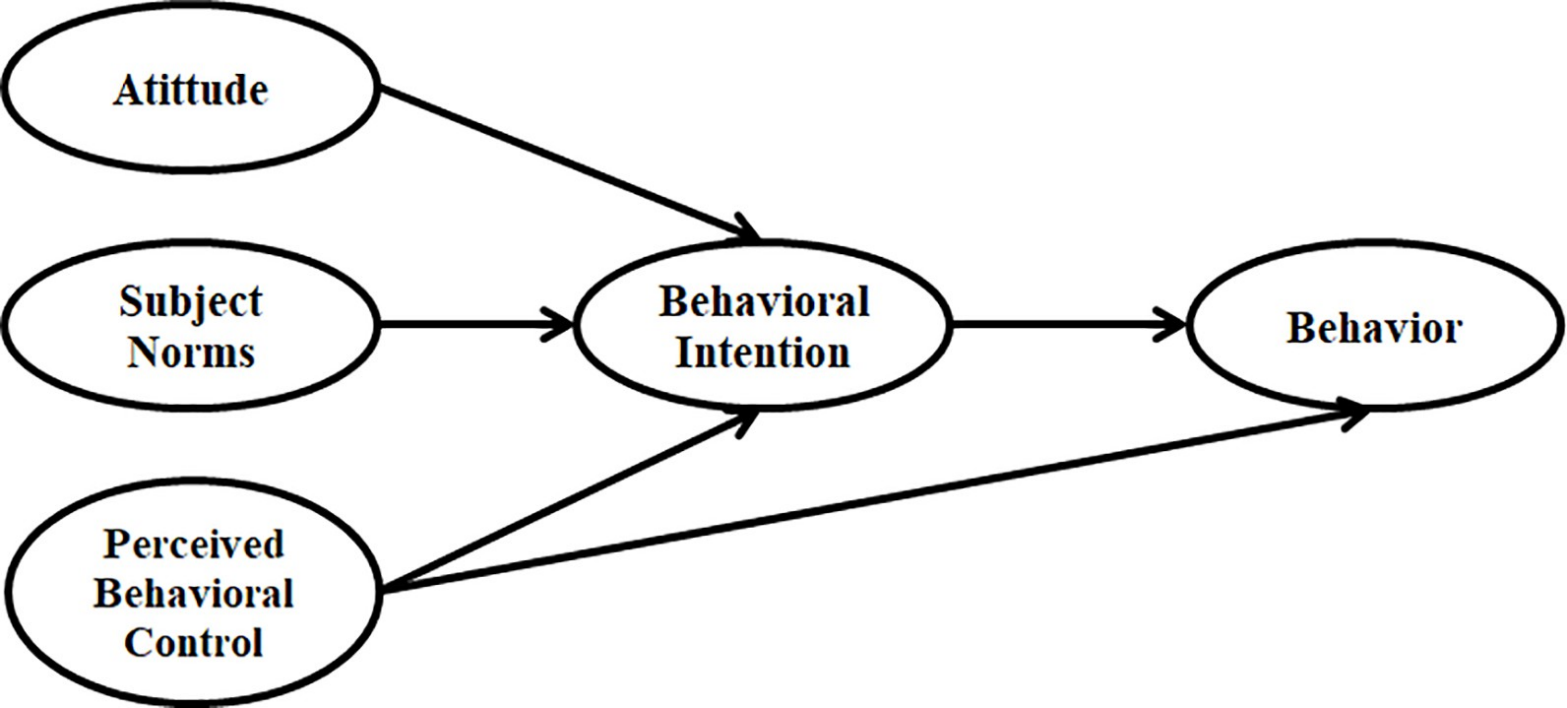
Theory of planned behavior framework [15].

C&DW is usually discarded in nature through an inadequate procedure, sometimes polluting natural resources. This fact is already an aspect that has been causing significant problems for metropolises worldwide [13]. Therefore, integrating influential behavioural and social factors can turn into essential characteristics in increasing the overall effectiveness and efficiency of the C&DW management system. Recently, human factors are considered a hot topic in C&DW management because stakeholders and workers’ attitudes, behaviour, and Intention could remarkably influence C&DW management practices [14]. In this regard, research scholars have been using the Theory of Planned Behavior (TPB) based on Ajzen [15] study as the main framework (Fig 1), and they extended this theory based on their research objectives.

**Fig 2:**
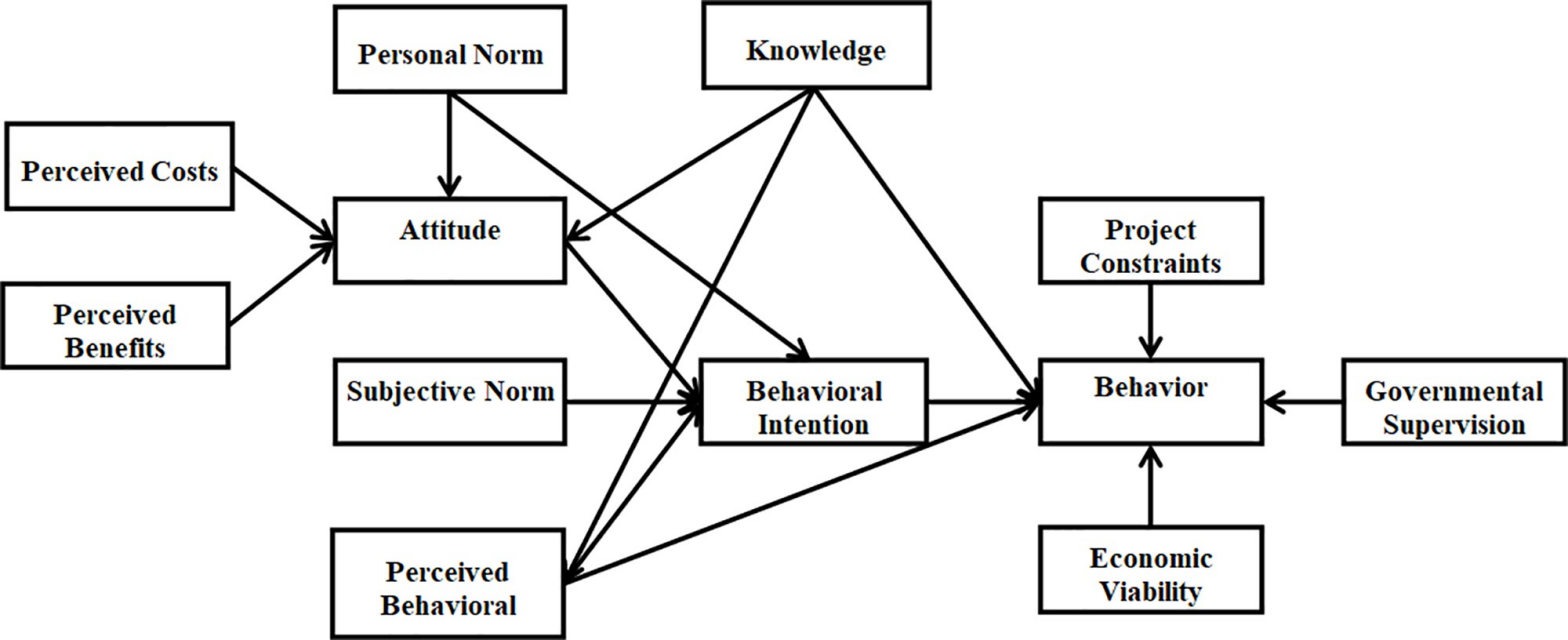
Extended TPB in this study’s research framework.

The first aim of this study is to analyze and develop an Extended TPB by combining three studies Li, Zuo [16], Wu, Ann [17], and Jain, Singhal [18]. Fig 2 present the C&DW management with Extended TPB.

Regression (multivariate or bivariate), fuzzy approaches, and dynamic system modelling are the most common statistical methods for analysing C&DW management with TPB. In recent decades, the trend in modelling TPB for CD&W management with structural equation modelling (SEM) technique grows [19–21]. Prior studies present seven estimators associated with the SEM statistical technique. Most SEM related articles applied maximum likelihood (ML) estimator in their analysis. Nevertheless, the ML estimator can be compromised by model misspecification. For example, Cole, Ciesla [22] noted that models with ML estimators are too strict with zero residual correlations, and exact zero cross-loadings may generate unfortunate model fitting results. Moreover, Asparouhov and Muthén [23] and Kolenikov [24] verified that the ML estimator brings significance parameter bias in factor loadings and factor correlations. Jenatabadi, Moghavvemi [25] determines the main disadvantages of SEM with the ML estimator. They observed that multivariate normal distribution of predicted (independent) variables and small sample size encouraged research scholars to find better SEM analysis approaches. The C&DW management with TPB or Extended TPB lacks deep analysis of SEM processes on multivariate normal distribution, presenting accuracy, and ML estimator comparison with other approaches. Significantly, a more accurate estimator can produce higher R-square and lower error indices for predicting dependent variables. The correct estimators can also reveal the relationship between research variables into non-significant and vice versa. Therefore, the second aim of this study compares different SEM estimators towards lower error terms and improved relationships among research variables.

## Theoretical background

### Extended theory of planned behaviour

Several theories have been proposed, refined, and implemented to better comprehend the connection between different facets of behavior and increased pro-environmental action. The most extensively used theory to forecast environmentally friendly behavior at the individual level is the theory of planned behavior (TPB). TPB is a social psychological model that explains how individuals’ attitudes, subjective norms, and perceived Behavioral control influence their intentions and behaviours. The TPB provides a framework for investigating behavioural choice determinants. Individual behaviour, according to the TPB, is the result of behavioural intentions, whereas intentions are a function of attitude towards the behaviour, subjective norms, and perceived behavioural control [26]. The TPB fundamentally asserts that the larger the behavioural intentions, the greater the likelihood that a given behaviour would be enacted. Despite widespread support, the approach has attracted a number of criticisms. The main complaint is that it needs to include more variables to improve its predictive and explanatory power. It is suggested that the TPB framework does not account for a large enough share of the variety in intentions. Ajzen [27] noted that the TPB allows for the incorporation of extra factors if they help significantly to understanding behaviour. As a result, several academics have proposed introducing new variables that are meaningful in the sense that they may theoretically influence intentions and behaviour in order to increase the TPB’s explanatory power. TPB associated with construction and demolition wate management behaviours have been expanded by researchers by adding more variables to improve the predictive accuracy of TPB such as social value [26], awareness of consequence [19], institutional pressures [18], governmental supervision [17], and concern for the community [14]. Our investigations build a conceptual model and give empirical data to authenticate the suggested model in the context of C&DW Recycle Behavior (as dependent variable) to strengthen the forecasting potential of the recycling behaviour model.

### Theory of the Bayesian and Bayesian SEM approach

**Fig 3:**
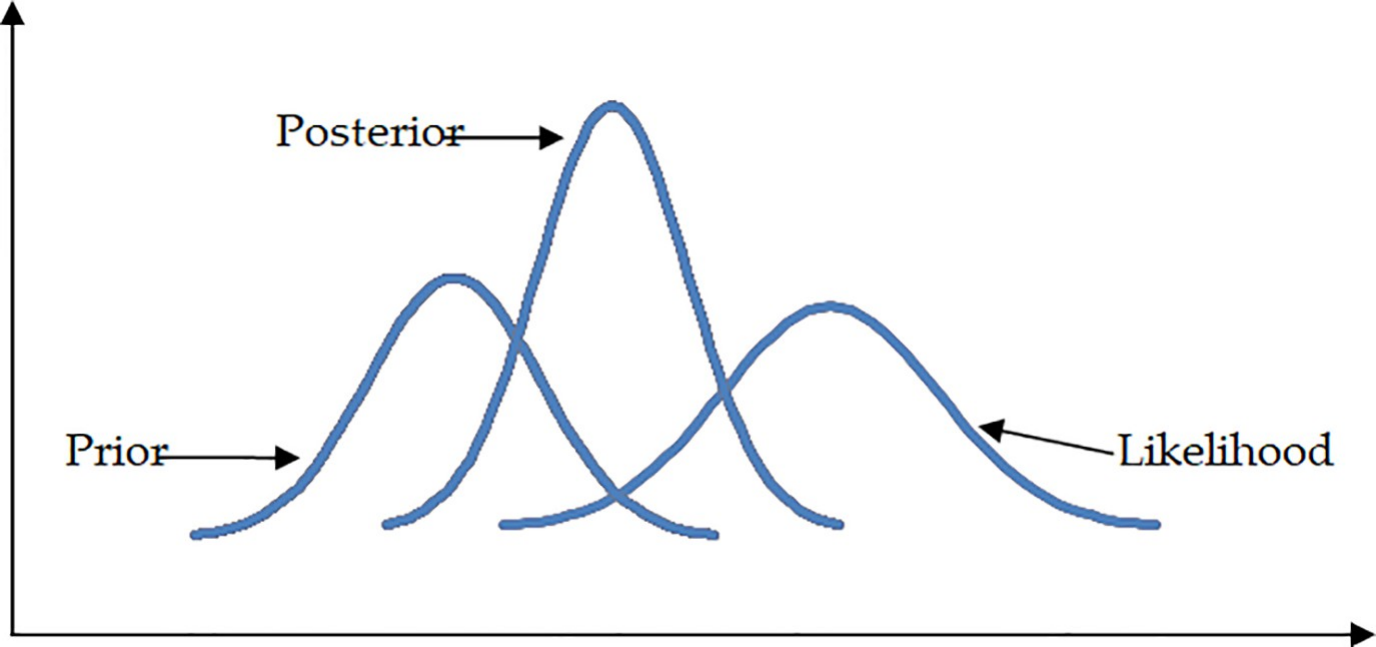
Likelihood, posterior and prior for a parameter (source: [29]).

In Bayesian analysis, the practitioner or expert has a preconceived notion, belief, or piece of information about the unknown parameter prior to observing the data. This prior knowledge is then updated with the information that was collected from the sample, which results in the construction of the posterior distribution of. This posterior distribution of will then be utilized to estimate. The steps involved in this process are laid out in Fig 3, which also provides an illustration of the likelihood as well as the distributions for a prior and its related posterior for a particular parameter. It is important to keep in mind that the likelihood can be thought of as the distribution of the data given the values of the parameters. According to Fig 3, the vast majority of the prior distribution contains parameter values that are lower than those found at the highest point of the likelihood. The posterior is the result of making concessions to both the prior and the likelihood in order to arrive at a conclusion.

It is clear from looking at Fig 3 that the prior does not assign a sufficient probability to locations where the likelihood is high, and that this results in a conflict between the prior and the data. For further information, please refer to Evans and Moshonov [28].

Priors might provide useful information or they might not. A non-informative prior, also known as a diffuse prior, may, for example, have a normal or uniform distribution with a wide variance. This type of prior is also known as a fuzzy prior. When using statistical modelling, a large variance is used to represent a significant amount of uncertainty in the parameter value. As a consequence of this, when there is a significant prior variance, the likelihood provides comparatively more information to the generation of the posterior, and the estimate becomes closer to an ML estimate. Evans [30] issued a warning that the Jeffreys-Lindley paradox might result from making use of a significant prior variance.

Formally speaking, Bayes’s theorem is used in the process of forming a posterior. Take into consideration the probabilities of the events *C* and *D*, denoted by *C* and *D*, *P*_*r*_ (*C*)and *P*_*r*_ (*D*), respectively. According to the theory of probability, the probabilities of *C* and *D* occurring together can be broken down into two categories: conditional and marginal.


$$P_{r}\left(C,D\right)=P_{r}\left(C|D\right)\;P_{r}\left(D\right)=P_{r}\left(D|C\right)P_{r}\left(C\right)$$ (1)

In Eq 1 can divide every side by $P_{r}(C)$ then

$$P_{r}\left(D|C\right)=\;\frac{P_{r}\left(C|D\right)\;P_{r}\left(D\right)}{P_{r}\left(C\right)}$$ (2)

It is commonly referred to as Bayes’ theorem. When modelling is done using this theorem, it allows the data x to play the role of C, and it allows the parameter value to play the role of *D*. As a result, the posterior region can be represented metaphorically as

posterior=parametergivendata

$$\begin{array}{l} =\;\frac{data|parameters\;\times parameters}{data}\\ =\;\frac{likelihood\;\times prior}{data} \end{array}$$

To be more exact, this is what we have.

$$P\left(\theta |x\right)=\frac{L\left(\theta \right)\pi \left(\theta \right)}{m\left(x\right)}$$ (3)

where *π*(*θ*) is the prior distribution (probability) of *θ* ∈ Ω and *m*(*x*) is called the prior predictive distribution of *x* obtains as (for continuous case)

$$m\left(x\right)=\int_{\Omega }L\left(\theta \right)\pi \left(\theta \right)d\theta$$ (4)

In SEM with Bayesian estimator, consider ***X*** is an independent and ***Y*** is a dependent latent vector, vector with a categorical or continuous or latent structure, and **Ω** is the coefficient matrix of latent variables.

***Y***, ***X*** and **Ω** are defined with the following structure:

$$Y=\left[\begin{array}{c} y_{1}\\ y_{2}\\ \vdots \\ y_{n} \end{array}\right];X=\left[\begin{array}{c} x_{1}\\ x_{2}\\ \vdots \\ x_{n} \end{array}\right];\Omega =\left[\begin{array}{c} \omega_{1}\\ \omega_{2}\\ \vdots \\ \omega_{n} \end{array}\right]$$ (5)

In the above equation, ***X*** is the observed data, which is augmented with the latent data (***Y*, Ω**) in the posterior analysis. **Θ** = (***τ***, ***θ***, **Ω**) indicates the parameter space and ***θ*** = (**Φ**, **Λ**, **Λ**_*ω*_, **Ψ**_*δ*_, **Ψ**_*ε*_) represents the structural parameters.

Eq 4 shows the prior distribution:

$$\pi \left(\Theta \right)=\pi \left(\tau \right)\pi \left(\theta \right)\pi (\Omega |\tau ,\theta )$$ (6)

A diffuse prior can be adopted based on the categorical ordinal structure of thresholds. Specifically, Eq 6 contains a constant *c* as follows:

$$\pi \left(\tau \right)=c$$ (7)

$$x_{1}=c\;if\;\tau_{c}-1<y_{1}<\tau_{c},$$ (8)

In Eq 8, the instant is called the process of ***x***_1_.

Besides, to modify a subjective perspective, a natural conjugate prior can be expected for ***θ*** with the conditional structure in Eq 6.


$$\pi \left(\theta \right)=\pi \left(\Lambda |\Psi_{\varepsilon }\right)\pi \left(\Psi_{\varepsilon }\right)$$ (9)

Eq 8 represents the distribution of $\left(\Lambda_{k}|\psi_{\varepsilon k}^{-1}\right)$.


$$\left(\Lambda_{k}|\psi_{\varepsilon k}^{-1}\right)∼N\left(\Lambda_{0k},\psi_{\varepsilon k}H_{0yk}\right)$$ (10)

However, $\psi_{\varepsilon k}^{-1}$ in Eq 10 has a gamma distribution Γ(*α*_0*εk*_, *β*_0*εk*_).

Where,

1. Γ denotes the gamma distribution,
2. **Λ**_*k*_ is the *k*th row of **Λ**,
3. *ψ*_*εk*_ is the *k*th diagonal element of ***ψ***_*ε*_. In this part, an inverse-Wishart distribution is considered for **Φ**, which is specified by:

$$\Phi ∼W^{-1}\left(R_{0}^{-1},\rho_{0}\right)$$ (11)

Moreover, it is assumed that all hyper-parameters are known and *L*(**Θ**│***X*** = ***x***)*π*(**Θ**) is defined as a posterior distribution.

The Markov Chain Monte Carlo (MCMC) technique is applied to generate a sequence with a configuration of random observations, |***X*** = ***x***. Because computing the **Θ**|***X*** = ***x*** posterior distribution is complicated, in the next step of the Bayesian SEM procedure a convergence test of the research model parameters must be done.

A model diagnostic is implemented according to Yanuar, Ibrahim [31] theory. Time series diagrams are designed to graphically determine the accuracy of the research model parameters with different starting measures. Then a diagnosis is undertaken by tracing the diagrams [32, 33].

To evaluate the acceptability of the research model suggested in this study, which combines a measurement model and a structural model, the residual estimates versus latent variable estimates are calculated to provide information on the model fit. The residual estimates for the measurement model (${\hat{\varepsilon }}_{i}$) are presented in Eq 10.

$${\hat{\varepsilon }}_{i}=y_{i}-\hat{\Lambda }{\hat{\xi }}_{i},\;i=1,2,\ldots ,n,$$ (12)

Where $\hat{\Lambda }$ and ${\hat{\xi }}_{i}$ are Bayesian estimates evaluated with the MCMC method. The estimates of the residuals in the structural equation (${\hat{\delta }}_{i}$) are defined as follows:

$${\hat{\delta }}_{i}=\left(I-\hat{B}\right){\hat{\eta }}_{i}-\hat{\Gamma }{\hat{\xi }}_{i},\;i=1,2,\ldots ,n,$$ (13)

Where $\hat{B},{\hat{\eta }}_{i},\hat{\Gamma }$ and ${\hat{\xi }}_{i}$, which are estimated according to the Bayesian structure, are calculated by simulating the research observations from MCMC.

The measurement model is defined in Eq 12:

$$y_{i}=\Lambda \omega_{i}+\varepsilon_{i},\;i=1,2,\ldots ,n,$$ (14)

## Materials and methods

### Survey instrument

There are several concerns in preparing the questionnaire for this study. The first concern is avoiding symmetric bias in gathering data; thus, the questionnaire must be prepared attentively and carefully. In practical studies with self-report surveys, common method bias status is the primary concern. Podsakoff, MacKenzie [34] suggest following both procedural and statistical remedies in reducing common method bias. An iterative process to prepare the questionnaire before gathering data is required. Accepting the opinions of statistical and construction experts can validate the questionnaire. The questionnaire includes three main sections. The first section introduces this study and lists the construction and demolition factors used in the research. The second section focuses on participant demographics, including education level, age, work experience, and gender. The third section is about measuring the Extended TPB model. Five industry specialists from the construction sector and seven academicians (four in the building construction and three statisticians) are consulted in improving the questionnaire. Afterwards, the pilot study is carried out involving fifty participants. Composite reliability of all the research variables except Personal Norm and Governmental Supervision are found more than 0.701. The two measurements are improved with the assistance of professionals.

Extended TPB for this study includes twelve latent variables like Project Constraints, Economic Viability, Governmental Supervision, Behavioral Intention, Knowledge, Perceived Behavioral Control, Subject Norms, Attitude, Personal Norm, Perceived Benefit, Perceived Cost, and C&DW Management Behavior. Table 1 shows theoretical literature for measuring latent variables and the number of questions for every latent variable.

**Table 1:** Theoretical literature for measuring latent variables and number of questions.

Latent Variable	Number of questions	Theoretical resource
Attitude	5 questions	
Subject Norm	6 questions	
Perceived Behavioral Control	5 questions	
Behavioral Intention	4 questions	
C&DW Recycle Behavior	7 questions	
Project Constraints	6 questions	
Economic Viability	5 questions	
Governmental Supervision	4 questions	
Perceived Benefit	4 questions	
Perceived Cost	3 questions	
Personal Norm	2 questions	
Knowledge	4 questions	

Cross-sectional research is designed in this study, so data is gathered from the research population at only one point in time. There is some statistical process for calculating sample size. For the SEM technique, Hair et al. [35] suggest a well-organised sampling size calculation. Their sampling size depends on the number of latent variables and the number of factors or measurement variables inside the latent variables (See Table 2).

**Table 2:** Calculating of sample size based on Hair et al. [35] theory.

Sample Size estimation	Criteria from the Research Model
**100 individuals (participants)**	If the research framework contains 1) five or less latent variables; 2) each latent variable contains at least three indicators
**150 individuals (participants)**	If the research framework contains 1) seven or less latent variables; 2) each latent variable contains at least three indicators
**300 individuals (participants)**	If the research framework contains 1) seven or less latent variables; 2) each latent variable has less than three indicators
**500 individuals (participants)**	If the research framework contains 1) more than seven latent variables; 2) each latent variable has less than three indicators

The research framework includes twelve latent variables. Therefore, at least 500 participants are needed to have enough accuracy for modelling. Six hundred questionnaires were distributed to respondents working in Kuala Lumpur, Penang, and Johor construction districts. The three states have the highest population and builder companies in Malaysia. Five hundred thirty completed questionnaires were received, out of which 70 were incomplete. The respondent’s rate of sampling for this study is 88.3%. A power analysis performed with the G*Power software revealed that a minimum sample size of 387 participants is required for the study if the effect size is assumed to be 0.15, the alpha value is assumed to be 0.05, and the power is assumed to be 0.80. In this investigation, structural equation modelling (SEM) was used to analyse the gathered data and evaluate the proposed hypothetical structural model. Maximum likelihood estimation was calculated with the help of AMOS 21.0 software.

The demographic Information of the participants is mentioned in Table 3. In terms of general status in the Malaysian construction industry, female respondents are significantly fewer. More than 7% of the participants are working under the main contractor. Among the 530 participants, 41% have a high school education. Forty-four per cent of the participants were skilled workers, 30% were site managers, and around 15% were project managers.

**Table 3:** Demographic information.

Character		Number	Percentage
**Gender**			
	Male	497	93.8%
	Female	33	6.2%
**Age**			
	Less than 25	106	20.0%
	25–34	131	24.7%
	35–45	141	26.6%
	higher than 45	152	28.7%
**Education Level**			
	High school	211	39.8%
	Diploma	196	37.0%
	Undergraduate	100	18.9%
	Postgraduate	23	4.3%
**Work experience**			
	Less than 5 years	153	28.9%
	5–10 years	134	25.3%
	11–15 years	126	23.8%
	more than 15 years	117	22.1%
**Occupation**			
	Project managers	83	15.7%
	site managers	161	30.4%
	site foreman/supervisors	45	8.5%
	skilled workers	228	43.0%
	Others	13	2.5%
Contractor category			
	General contractor	405	76.4%
	subcontractor	125	23.6%

#### Ethics, consent, and permissions

The survey was conducted according to the University of Malaya’s Research Ethics Committee (UM.TNC2/RC/H&E/UMREC 127) approval. The research methods were performed following the relevant guidelines and regulations, and informed consent was obtained from all the respondents. Respondents were also provided with an explanation of the research purpose.

## Results

### Validity, reliability, and full collinearity assessment

The following statistical processes should be done for validity and reliability confirmation following Fornell and Larcker [36]:

1. Cronbach’s alpha is the most familiar statistical index for validity confirmation. Nunally and Bernstein [37] suggested that the value of this index should be greater than 0.7 for acceptancy of validity. However, Bagozzi and Yi [38] accept validity if coefficient α is higher than 0.60.
2. Average Variance Extracted (AVE) is the familiar index for reliability. Segars [39] suggested that the value of this index should be greater than 0.5 for reliability acceptance.

Table 4 shows the Cronbach’s alpha and AVE index values after some statistical processes in the pilot study. These indices meet the suggested ideals and norms. Therefore, the validity and reliability of the research model are accepted.

**Table 4:** AVE and Cronbach’s Alpha indices for validity and reliability analysis.

Latent Variables	Cronbach’s Alpha	AVE	VIF
Perceived Benefit	0.701	0.555	[1.67, 2.45]
Perceived Cost	0.712	0.562	[2.33, 3.46]
Personal Norm	0.707	0.602	[1.88, 3.87]
Knowledge	0.745	0.721	[2.04, 3.66]
Perceived Behavioral Control	0.802	0.592	[2.22, 3.88]
Attitude	0.818	0.612	[2.87, 3.76]
Subjective Norm	0.734	0.576	[3.11, 4.07]
Behavioral Intention	0.767	0.672	[2.04, 3.16]
C&DW Recycle Behavior	0.721	0.818	[1.34, 2.45]
Governmental Supervision	0.801	0.652	[2.45, 3.67]
Economic Viability	0.722	0.709	[2.33, 3.66]
Project Constraints	0.759	0.717	[1.89, 3.89]

Before assessing the structural model, it is necessary to ensure that there is no linear relationship between the elements. According to Hair, Risher [40], variation inflation factor (VIF) values less than 5 were acceptable.

In the last stage of measurement model evaluation, discriminant validity was evaluated. As suggested by Hair Jr, Sarstedt [41], the heterotrait–monotrait ratio (HTMT) is the most rigorous and effective test for assessing the discriminant validity, which was previously evaluated using the Cross-Loadings and Fornell-Larcker criterion tests. The HTMT values were determined using complete bootstrapping with 5000 iterations. Our results demonstrated that every HTMT value was less than 0.85; consequently, the discriminant validity was accepted.

### Structural model

For structural model we compared four types prior input. To achieve this, the models with four types of prior inputs are compared. Lee [42] suggested to assign values to the hyperparameters, a minor variance is considered to each parameter as. We determined four prior inputs are considered correspondingly as follows:

Hyperparameter β_1_ is the effect of Behavioral Intention on C&DWM Behavior
Hyperparameter β_2_ is the effect of Knowledge on C&DWM Behavior
Hyperparameter β_3_ is the effect of Perceived Behavioural Control on C&DWM Behavior
Hyperparameter β_4_ is the effect of Project Constraint on C&DWM Behavior
Hyperparameter β_5_ is the effect of Governmental Supervision on C&DWM Behavior
Hyperparameter β_6_ is the effect of Economic Viability on C&DWM Behavior
Type I Prior: loadings values were introduced equal to 0.5. The measures corresponding to {*β*_1_, *β*_2_, *β*_3_, *β*_4_, *β*_5_, *β*_6_} are {0.6, 0.6, 0.5, 0.7, 0.6, 0.7}.
Type II Prior: Every hyperparameter are equal half of the values in Prior I
Type III Prior: Every hyperparameter are equal a quarter of the values in Prior I
Type IV Prior: Every hyperparameter are equal a double the values in Prior I

Table 5 shows the outputs of four prior input types. It can be confirmed that the indices found in terms of SEM with Bayesian estimator processes are not sensitive to these four prior inputs. Accordingly, in discussing the outputs obtained involving Bayesian SEM, the results obtained with the type I prior are used.

**Table 5:** Estimated parameter with standard errors for four types of prior distribution.

Parameter	Type I Prior		Type II Prior		Type III Prior		Type IV Prior	
	Estimate	STD	Estimate	STD	Estimate	STD	Estimate	STD
β_1_	0.462	0.489	0.532	0.567	0.422	0.509	0.444	0.491
β_2_	0.665	0.379	0.698	0.456	0.661	0.469	0.521	0.399
β_3_	0.178	0.567	0.169	0.647	0.157	0.731	0.122	0.605
β_4_	0.065	0.345	0.033	0.415	0.051	0.429	0.032	0.346
Β_5_	0.194	0.234	0.111	0.333	0.146	0.335	0.199	0.239
Β_6_	0.551	0.278	0.456	0.498	0.501	0.666	0.521	0.311

**Fig 4:**
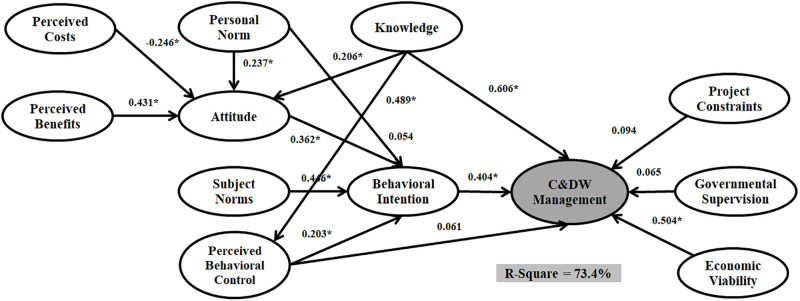
The structural model with PLS estimator.

**Fig 5:**
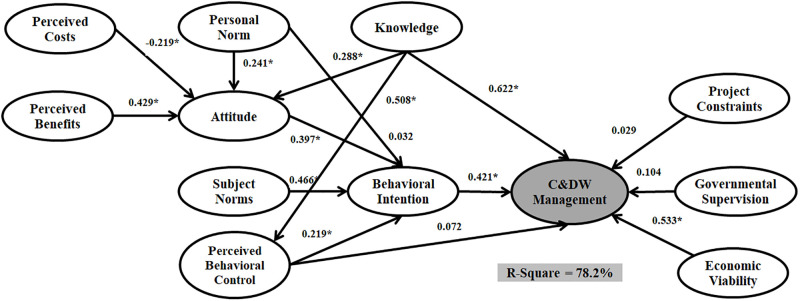
The structural model with an ML estimator.

**Fig 6:**
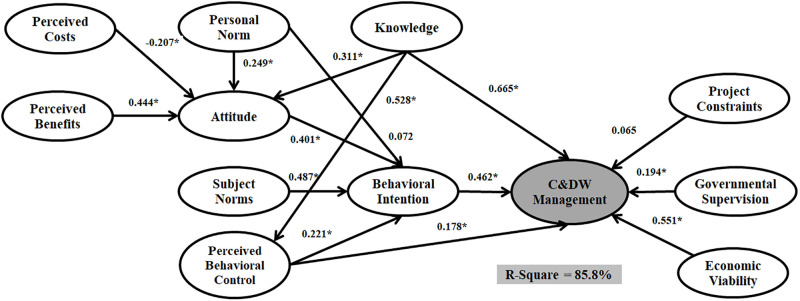
The structural model with a Bayesian estimator.

The data were run through three estimators, PLS, ML, and Bayesian. Figs 4–6 and Table 6 shows the models outputs. The significance levels of paths indicated whether or not the structure model of SEM analysis approves the hypothetical association. Table 6 present the estimated structural equations that address the relationships among twelve latent variables for SEM_PLS_, SEM_ML_, and SEM_Bayesian_. Among fifteen hypotheses, eleven were accepted from both SEM_PLS_ and SEM_ML_ analyses, and thirteen were accepted based on the SEM_Bayesian_ technique.

**Table 6:** Structural model among three estimators.

Relationship		PLS	ML	Bayesian
H1	Attitude **→** Behavioral Intention	0.362*	0.397*	0.401*
H2	Subject Norms **→** Behavioral Intention	0.446*	0.466*	0.487*
H3	Perceived Behavioral Control **→** Behavioral Intention	0.203*	0.219*	0.221*
***H4***	*Perceived Behavioral Control → C&DW Management Behavior*	***0*.*061***	***0*.*072***	*0*.178*
H5	Behavioral Intention **→** C&DW Management Behavior	0.404*	0.421*	0.462*
H6	Perceived Costs **→** Attitude	-0.246*	-0.219*	-0.207*
H7	Perceived Benefits **→** Attitude	0.431*	0.429*	0.444*
H8	Personal Norm **→** Attitude	0.237*	0.241*	0.249*
H9	Personal Norm **→** Behavioral Intention	0.054	0.032	0.072
H10	Knowledge **→** Attitude	0.206*	0.288*	0.311*
H11	Knowledge **→** Perceived Behavioral Control	0.489*	0.508*	0.528*
H12	Knowledge **→** C&DW Management Behavior	0.606*	0.622*	0.665*
H13	Project Constraints → C&DW Management Behavior	0.094	0.029	0.065
***H14***	***Governmental Supervision → C&DW Management Behavior***	***0*.*065***	***0*.*104***	*0*.194*
H15	Economic Viability **→** C&DW Recycle Behavior	0.504*	0.533*	0.551*

According to the results, Personal Norm, Knowledge, and Perceived Costs significantly affect the Attitude for the three models. Moreover, Perceived Behavioral Control and Attitude have a significant effect on Behavioral Intention for the three models. However, Subject Norm does not significantly affect Behavioral Intention for both SEM_ML_ and SEM_PLS_, while there is a significant impact for SEM_Bayesian_. There is no significant relationship between Perceived Behavioral Control and C&DW Management Behavior for SEM with ML and PLS estimators. However, there is a significant relationship between Perceived Behavioral Control and C&DW Management Behavior in SEM outputs.

A comparison of the three structural models Implies that the SEM_Bayesian_ estimator outperforms both SEM_ML_ and SEM_PLS_. SEM_ML_ and SEM_PLS_ can predict 73.4% and 78.2% of the variance in C&DW Management Behavior, while SEMBayesian predicted 85.8%. Moreover, the regression coefficient for all relationships (beta value) was stronger in the model assumed with SEM_Bayesian_. These results approve the superior capability of SEM_Bayesian_ over the other SEM_ML_ and SEM_PLS_ techniques.

The first group of additional variables are Knowledge and Personal Norm. Three SEM outputs confirmed that Knowledge has the highest direct effect on C&DW Management Behavior (SEM_PLS_ = 0.606, SEM_ML_ = 0.622, and SEM_Bayesian_ = 0.665), followed by Perceived Behavioral Control (SEM_ML_ = 0.376, SEM_PLS_ = 0.333, and SEM_Bayesian_ = 0.428) and Attitude (SEM_ML_ = 0.206, SEM_PLS_ = 0.288, and SEM_Bayesian_ = 0.311). Personal Norm had a relatively weak direct effect on Behavioral Intention (SEM_PLS_ = 0.054, SEM_ML_ = 0.032, and SEM_Bayesian_ = 0.072) but significant impact on Attitude (SEM_PLS_ = 0.237, SEM_ML_ = 0.301, and SEM_Bayesian_ = 0.249). The second additional group of variables are Perceived Benefits and Perceived Costs. Table 6 presents that Perceived Costs has a significant negative effect on Attitude and that Perceived Benefits has a positive but not significant effect on Attitude. The fourth additional group of variables are the Project Constraints, Economic Viability, and Governmental Supervision. Interestingly, these variables have a significant effect on C&DW Management Behavior. Project Constraints has the highest impact (SEM_PLS_ = 0.424, SEM_ML_ = 0.499, and SEM_Bayesian_ = 0.565) while Governmental Supervision has no significant effect on C&DW Management Behavior. The Economic Viability variable has no significant impact on the Bayesian and PLS estimators. However, this relationship is significant on C&DW Management Behavior when applying the ML estimator (SEM_PLS_ = 0.004, SEM_ML_ = 0.129, and SEM_Bayesian_ = 0.051).

### Comparison analysis among SEM_ML_, SEM_PLS_, and SEM_Bayesian_

This section compares the SEM_ML_, SEM_PLS_, and SEM_Bayesian_ outputs in estimating C&DW Management Behavior in the extended TPB structure. Four leading indices were involved to compare the three types of estimators: coefficient of determination (R-Square; R^2^), mean absolute error (MSE), root mean square error (RMSE), and mean absolute percentage error (MAPE). They are defined with the following equations:

$$\text{Coefficient}\;\text{of}\;\text{determination}=\text{R}-\text{Square}=\;\frac{{\left[\sum_{i=1}^{n}\left(y_{i}^{,}-\;{\bar{y}}_{i}^{,}\right).\;\;\left(y_{i}-\;{\bar{y}}_{i}\right)\right]}^{2}}{\sum_{i=1}^{n}\left(y_{i}^{,}-\;{\bar{y}}_{i}^{,}\right).\;\;\sum_{i=1}^{n}\left(y_{i}-\;{\bar{y}}_{i}\right)}$$ (15)

$$\text{Root}\;\text{mean}\;\text{square}\;\text{error}\;\left(\text{RMSE}\right)=\;\sqrt[{2}]{\frac{\sum_{i=1}^{n}{\left(y_{i}^{,}-y_{i}\right)}^{2}}{n},}$$ (16)

$$\text{Mean}\;\text{absolute}\;\text{error}\;\left(\text{MSE}\right)=\;\frac{\sum_{i=1}^{n}\left|y_{i}^{,}-y_{i}\right|}{n}$$ (17)

$$\text{Mean}\;\text{absolute}\;\text{percentage}\;\left(\text{MAPE}\right)=\;\frac{1}{n}\;\sum_{i=1}^{n}\left|\frac{y_{i}^{,}-y_{i}}{y_{i}}\right|$$ (18)

In the Eqs 15–18, *y*_*i*_ is the *i*th real value of the dependent variable (C&DW Management Behavior) and *y*_*i*_, is the *i*th predicted value. Table 7 reveals the values of the four statistical indices, containing R-Square, RMSE, MSE and MAPE for SEM with three different estimators.

**Table 7:** Comparison analysis among SEM_PLS_, SEM_ML_ and SEM_Bayesian_.

	SEM Statistical Indices			
	R-Square	RMSE	MSE	MAPE
SEM_PLS_	0.734	0.177	0.287	0.095
SEM_ML_	0.782	0.102	0.136	0.077
SEM_Bayesian_	0.858	0.067	0.092	0.051

The R-Square for SEM_Bayesian_ is 0.858, meaning 85.8% of the variation in C&DW Management Behavior dependent variables were associated with eleven latent variables, i.e., Project Constraints, Economic Viability, Governmental Supervision, Behavioral Intention, Knowledge, Perceived Behavioral Control, Subject Norms, Attitude, Personal Norm, Perceived Benefit, and Perceived Cost. The R-Square for SEM_ML_ is 78.2%, and SEM_PLS_ is equal to 73.4%. The R-Square of SEM_Bayesian_ is higher than both SEM_ML_ and SEM_PLS_. This result indicates that the association among independent and dependent variables in SEM_Bayesian_ is stronger than in SEM_PLS_ and SEM_ML_. As a result, R-Square demonstrates the model goodness-of-fit and proves our data is more fitting to SEM_Byesian_ than SEM_PLS_ and SEM_ML_.

In terms of error indices mentioned in Eqs 2–4, the mean absolute percentage error, root mean squared error, and mean absolute error values for the SEM_Bayesian_ outputs (0.051; 0.067; 0.092) are lower than for SEM_PLS_ (0.095; 0.177; 0.287) and SEM_ML_ (0.077; 0.102; 0.136). Subsequently, applying the SEM technique with Bayesian estimator benefits the Extended TPB compares to the ML and PLS estimators.

## Discussion

The purpose of this article was to research and present a better understanding of SCDWM in the Chinese construction sector. This was accomplished by investigating the many indications of each contributing element to C&DWM and determining whether or not these factors may potentially effect C&DW Management Behavior.

### Comparison with previous studies

Our findings are consistent with several recent efforts that have been involved in the overarching topic of C&DW Management Behavior. The builders’ attitudes and their sense of how much behavioral control they have are examples of the internal motivational elements. Builders have a favorable attitude towards C&DW recycling as a result of the perception that the benefits received from recycled items outweigh the expenses associated with using them. This finding is in line with the findings that were found in previous studies [14, 19, 20, 43]. It was discovered that attitude (H1) and perceived behavioral control (H3) both had a beneficial influence on the behavioral intention of C&DW recycling. These findings are consistent with the research that has been conducted previously [16, 26, 44]. People’s opinions of how easy or difficult it is to carry out a specific behavior are connected to their level of perceived behavioral control.

Individual-centric factors play a significant role in determining both the perceived behavioral control of builders and their attitudes toward their work. These findings suggest that perceived behavioral control and attitude could be two of the important skills that could be assessed for individuals assigned to roles involved in decision making related to C&DW in businesses in the real estate and infrastructure sectors that are looking to increase C&DW recycling. Our observation of Subjective Norm differs from other studies like Jain, Singhal [18], and Li, Tam [45]. Their findings may be influenced by low social awareness regarding C&DW Management in India and Shenzhen, China. Low awareness in C&DW Management is associated with social norms and low societal expectations. Social norms and beliefs can change as societal awareness grows over time or due to governments’ supplying sufficient information to the public. Subjective Norm can emerge as a positive relation with Behavior Intention in C&DW Management. Meanwhile, applying a more accurate predictor can potentially generate different outputs, requiring us to adopt different strategies.

Perceived Benefits derived from C&DW products exceeds their Perceived Costs, resulting in a positive Attitude of construction workers towards C&DW management. The construction workers for infrastructure companies are always looking to C&DW management to optimize recycling. The outputs of the Extended TPB with SEM_Bayesian_ reveal that both Perceived Benefits and Perceived Costs can be the two deciding indicators when builders consider C&DW management. These determinations are consistently supported by previous studies [18, 26].

In terms of sustainability, the outputs of this research resonate with several recent studies in extended TPB with SEM. Furthermore, statistical modelling can provide useful insights into finding better estimators based on data structure. Otherwise, researchers may face less accurate analysis and misleading policy and decision-makers. Attitude, Subject Norms, and Perceived Behavioral Control are the main predictors in TPB studies. We have two main concerns about these three variables in our research model. The first issue is concerned with the effects of attitude and perceived control behaviour. Figs 4–6 and Table 6 show the outputs of SEM with PLS, ML, and Bayesian estimators. We found that both Attitude (H1) and Perceived Behavioral Control (H3) have a significant positive impact on Behavioral Intention, which complements prior studies by [16] and [26]. Individual-centric and critical elements distinguish construction workers’ perceived behavioural control and attitude. Perceived Behavioral Control is related to people’s sensitivities about the effortlessness or uneasiness of a specific behaviour. The second concern is the situation of Subjective Norm in our research model. Following Table 6, Subjective Norm does not significantly influence Behavioural Intention with both ML and PLS estimators. However, Subjective Norm has a significant and positive influence on Behavioral Intention when applying the Bayesian estimator.

In our study, the effect of Personal Norm on Behavioral Intention is rejected. Table 6 shows Personal Norm has a significant indirect effect on Behavioural Intention through Attitude (0.249×0.401 = 0.10). Personal Norm’s impact is relatively insignificant than Knowledge or even the original TPB variable, the Subjective Norm, scoring (0.249×0.401+0.072 = 0.172) on Behavioural Intention and (0.072×0.462 = 0.033) on C&DW Management Behavior. The implication is that internal moral norms do not impact C&DW Management Behavior as what was supposed to be. This result is conflicting with the output in Tonglet, Phillips [46]. The argument is that the predictor Attitude has partially combined the impacts of the Personal Norm following Kaiser [47]. If an individual has a sturdy moral norm, he/she correspondingly has a positive attitude on C&DW Management Behavior. On the other hand, the external social norm is more instrumental and significant than the internal moral norm on C&DW Management Behavior. Our output reflects this finding where we observe that the total effect of Subjective Norm (0.234) is far higher than the Personal Norm (0.002). This condition is not hard to understand. Considering Malaysia as a developing country, many Malaysian are still struggling to have a better quality of life. The situation is more sensitive for construction workers, who have lower salaries. Therefore, it is not surprising that the Malaysian contractor employees present more concern about the pressure from their clients, line managers, and even colleagues than their consideration about C&DW.

The three independent variables of original TPB, Attitude, Subjective Norm, and Perceived Behavioral Control, all significantly affected Behaviour Intention in this study. Moreover, Behavior Intention has a significant impact on C&DW Management Behavior. The SEM_ML_ application supports Perceived Behavioral Control impacts on C&DW Management Behavior. Nevertheless, when the SEM_Bayesian_ is applied, the hypothesis related to the impact of Perceived Behavioral Control on C&DW Management Behaviour is not in agreement. Our findings show that the three independent variables impact C&DW Management Behavior only through Behavioral Intention. The result contrasts the conclusions in Jain, Singhal [18], Wu, Ann [17], and Zhu and Li [48]. In their studies, Perceived Behavioral Control was the only direct determinant of C&DW Management Behavior. The varying roles of the three TPB predictors may be subsidized the firmer protocols and progression of construction technology in Malaysia. The regulations and guidelines must extend to owners, contractors, and even designers to ensure recycling and construction waste reduction. These days, construction and builders monitor material waste and progressively implement precast concrete elements and metal formwork. Despite that, Knowledge becomes the highest significant variable to the C&DW Management Behavior in the Extended TPB (see Table 6). The Knowledge effect (0.655) is far greater than the main three predictors of Attitude (0.401), Subjective Norms (0.487), and Perceived Behavioral Control (0.211). The results show that the C&DW Management Behavior believes the constructor employee is competent of their knowledge of C&DW Management. Furthermore, Knowledge indirectly affects C&DW Management Behavior via Attitude (0.057) and Perceived Behavioural Control (0.094). The total effect of Knowledge on C&DW Management Behavior is 0.816, demonstrating that Knowledge determines the implementation of C&DW Management Behavior directly rather than indirectly via other variables. As a result, the most effective way to develop C&DW Management Behavior is to train and educate construction employees. These outputs are reflected in Li, Zuo [16], and Wang, Kang [49].

The original TPB framework provides a fundamental framework for recognizing C&DW Management Behavior. In comparison, the Extended TPB enhances the C&DW Management Behavior. The R-Square of the Extended TPB model (0.858) is 2.077 times as big as the original TPB model (0.413). The improvement signifies that the additional variables following Jain, Singhal [18], Li, Zuo [16], and Wu, Ann [17] made a prominent contribution in developing the explanation power of the Extended TPB model. The explanation power can be increased by including other vital research variables.

The SEM_ML_ and SEM_PLS_ represent parametric modelling, while the SEM_Bayesian_ can be considered a semi-parametric modelling approach. Figs 4–6 and Table 7 describe the SEM_Bayesian_, SEM_ML_, and SEM_PLS_ outputs. The R-Square value for SEM_Bayesian_ (85.8%) is greater than SEM_ML_ (78.2%) and SEM_PLS_ (73.4%) analyses. Governmental Supervision and Perceived Behavioral Control positively impact C&DW Management Behavior following the Bayesian estimator adoption. Nevertheless, the relations within the PLS and ML estimators were not significant. We also show that applying a more accurate estimator to the SEM technique improves the relationship among research variables in Extended TPB modelling and substantially changes policymakers’ decisions.

### Practical implications

There are two managerial implications from our outputs. First, Subjective Norm confirms a stimulating effect on optimizing C&DW management Behavior, particularly on Behavior Intention. For this reason, more stringent protocols must be considered to address this issue. The pressure from public concerns and stringent rules and regulations can encourage and leverage builders’ attention to C&DW management. This pressure can be transmitted to contractor employees through customers, enterprise administrators, and their colleagues, therefore supporting construction waste reduction performance. Second, for estimating C&DW Management Behavior, we define Knowledge as the most significant influential variable in this research. Addressing this matter is a priority in upgrading construction contractors’ and employees’ knowledge of waste reduction.

### Theoretical implications

Examining Extended TPB through the Bayesian, ML, and PLS estimators proves that the Bayesian estimator produced more accurate outputs in estimating the number of sound parameters. Moreover, error indices (RMSE, MSE, and MPE) demonstrate that SEM with a Bayesian estimator provides a practically well-fitted model with a higher Coefficient of Determination [R-Square; R^2^] (0.858) than both SEM_ML_ (0.782) and SEM_PLS_ (0.734). The proposed Bayesian estimator decreases the MSE from 0.136_ML_ and 0.287_PLS_ to 0.092_Bayesian_, RMSE from 0.102_ML_ and 0.177_PLS_ to 0.067_Bayesian_, and MPE from 0.077_ML_ and 0.095_PLS_ to 0.051_Bayesian_ (see Table 7). The SEM_Bayesian_ values are predicted to be closer to the actual values than both SEM_ML_ and SEM_PLS_. From a theoretical point of view, this study shows that a Bayesian estimator can give better results when testing Extended TPB and predicting C&DW Management Behaviour.

### The novelty of this study

The first thing that makes this research unique is that it uses Bayesian Structural Equation Modelling analysis to understand behavioural intention in C&DW management for the very first time. This is the first time that this has been done.

The idea of planned behaviour is extended in this study by drawing on three different theories that came before it, which brings us to the second original aspect of this research.

The vast bulk of earlier research that was conducted on behavioural intention was mostly dependent on variables that were established by established theories. The originality of this research lies in the fact that actual behaviour restrictions, such as project constraints, economic feasibility, and governmental supervision, are included into the core idea of planned behaviour model in order to increase its scope.

## Conclusion

The research framework proposed combines three studies [16–18] related to Extended TPB in construction C&DW management and recommends Bayesian SEM. Such a comprehensive framework delivers improved perceptions of Perceived Cost, Perceived Benefit, Personal Norm, knowledge, Governmental Supervision, Economic Viability, and Project Constraints, as well as individual behaviour towards resource efficiency and sustainability. Visions and understandings from the study will help the regulators and government establish better strategies towards sustainable C&DW management in Malaysia. The outputs also benefit the private construction sector by realizing the significance of several drivers of C&DW Management Behavior.
